# Evaluation of Systemic Lutein Supplementation on Periodontal Repair in Experimental Periodontitis in Wistar Rats

**DOI:** 10.3390/dj14080466

**Published:** 2026-08-02

**Authors:** Otávio Augusto Pacheco Vitória, Vivian Cristina Noronha Novaes, Elisa Mara de Abreu Furquim, Henrique Rinaldi Matheus, Bianca Rafaeli Piovezan, Luiz Guilherme Fiorin, Ruan Henrique Delmonica Barra, Gabriela Carrara Simionato, Ester Oliveira Santos, Edilson Ervolino, Juliano Milanezi de Almeida

**Affiliations:** 1 Periodontics Division, Department of Diagnostic and Surgery, School of Dentistry, São Paulo State University (UNESP), Araçatuba 16015-050, SP, Brazil; otavio.pacheco@unesp.br (O.A.P.V.); elisa.furquim@unesp.br (E.M.d.A.F.); hrmatheus@usp.br (H.R.M.); bianca.piovezan@unesp.br (B.R.P.); guilherme@fior.in (L.G.F.); ruan.barra@unesp.br (R.H.D.B.); gabriela.carrara@unesp.br (G.C.S.); ester.santos@unesp.br (E.O.S.); 2 Nucleus of Study and Research in Periodontics and Implantology (NEPPI), School of Dentistry, São Paulo State University (UNESP), Araçatuba 16015-050, SP, Brazil; vcnnovaes@hotmail.com (V.C.N.N.); e.ervolino@unesp.br (E.E.); 3 Department of Periodontics, University Center of Santa Fé do Sul (UNIFUNEC), Santa Fé do Sul 15775-000, SP, Brazil; 4 Division of Periodontology, College of Dentistry, University of São Paulo, São Paulo 05508-000, SP, Brazil; 5 Department of Basic Sciences, School of Dentistry, São Paulo State University (UNESP), Araçatuba 16018-805, SP, Brazil

**Keywords:** lutein, periodontal disease, inflammation, periodontitis, bone and bones

## Abstract

**Objectives:** The aim of this study was to evaluate the influence of systemic lutein (LT) supplementation on the progression of experimental periodontitis (EP) and its impact on mechanical periodontal therapy (SRP) in rats. **Methods:** A total of 120 male Wistar rats were used, and EP was induced by placing a ligature around the left mandibular first molar. The animals were allocated into four groups: NT (no treatment)/VEH (vehicle), NT/LT, SRP/VEH and SRP/LT. Supplementation was administered daily by oral gavage (0.7 mL/kg of vehicle or 250 mg/kg of LT). Animals were euthanized at 7, 15 and 30 days, and the hemimandibles were collected for histomorphometric analysis of the percentage of bone in the furcation region (PBF), histopathological assessment and immunohistochemical detection of inflammatory mediators (tumor necrosis factor-alpha, interleukin-1β and interleukin-10) and bone markers (tartrate-resistant acid phosphatase and osteocalcin). **Results:** Systemic LT supplementation, particularly when associated with scaling and root planning (SRP/LT), reduced the intensity and extent of the inflammatory infiltrate, partially decreased immunolabelling for IL-1β and TNF-α, and promoted greater organization of the connective tissue. Higher PBF values and fewer TRAP-positive cells were observed in LT-treated groups. **Conclusions:** Systemic LT supplementation reduced inflammation and alveolar bone resorption and enhanced the effects of mechanical periodontal therapy.

## 1. Introduction

Periodontitis is a chronic multifactorial disease that leads to the destruction of periodontal tissues through the perpetuation of a dysbiotic process within the oral microbiota, accompanied by an exacerbated host immune–inflammatory response. Individual susceptibility factors directly influence both the development and clinical manifestation of the disease [1,2,3,4]. Scaling and root planning (SRP) is the primary therapeutic approach for periodontitis, as it promotes periodontal tissue homeostasis by decontaminating the infected site, reducing the bacterial load, and facilitating tissue repair [5,6]. However, the effectiveness of SRP may be compromised when the host displays a persistent local immune–inflammatory response to the microbiome or when systemic adjunctive approaches, such as antimicrobials, fail due to bacterial resistance, thereby hindering the achievement of optimal periodontal therapy outcomes [7]. Consequently, alternative strategies administered systemically or locally have been proposed to enhance the predictability of periodontal treatment and modulate its destructive features, including the use of immunomodulatory agents [8,9,10]. In this context, phytotherapeutics represent a promising adjunctive modality when combined with SRP, given their capacity to modulate the host immune–inflammatory response and control disease progression [11]. The beneficial effects of several phytochemicals, such as green tea [12], curcumin [13], and propolis [14], have already been demonstrated in periodontitis.

Carotenoids (CAT) comprise a class of phytochemicals formed by isoprenoid molecules synthesized by plants and microorganisms, widely used in the prevention of chronic conditions, including ocular and cardiovascular diseases [15]. Evidence indicates that CAT contribute to increased bone density and protection against osteoporosis [16,17]. Within the context of periodontitis, the literature demonstrates that CAT exert beneficial effects on periodontal tissues by supporting tissue repair through immune–inflammatory modulation [18,19,20]. In addition, recent evidence has demonstrated an inverse association between systemic levels of carotenoid, particularly lutein, and the prevalence of periodontal disease, suggesting a potential protective role of these compounds in periodontal health [21]. Lutein (LT) is a xanthophyll carotenoid commonly used as a preventive agent for age-related macular degeneration and diabetic retinopathy due to its potent antioxidant and anti-inflammatory properties [22,23]. Studies have shown that LT downregulates the synthesis of pro-inflammatory mediators, including interleukin (IL)-1β, IL-6, IL-8, IL-12, and tumor necrosis factor-alpha (TNF-α), and attenuates activation of the nuclear factor kappa B (NF-κB) signaling pathway, which plays a central role in the expression of chemokines and cytokines associated with periodontal tissue damage [24]. Furthermore, evidence suggests that LT modulates bone metabolism by suppressing osteoclast differentiation in the presence of IL-1, promoting osteoblastic activity, and reducing sclerostin expression [25,26]. In addition, LT is frequently administered in combination with other antioxidant compounds, such as vitamins C and E or other CAT, which may exert synergistic effects in modulating oxidative stress and inflammatory responses [27,28]. However, evidence regarding such interactions specifically in the context of periodontal disease remains limited. Given that periodontitis is characterized by a close interplay between inflammatory mediators and bone metabolism, disease progression is driven by pro-inflammatory cytokines (e.g., TNF-α and IL-1β), whereas resolution and tissue repair involve regulatory cytokines (e.g., IL-10) and bone-forming activity (e.g., osteocalcin). Thus, modulation of inflammatory pathways may directly influence bone remodeling dynamics, linking the control of inflammation to the preservation of periodontal structures [1].

Collectively, these findings suggest that LT may act on key mechanisms involved in the pathogenesis of periodontitis. However, to date, no studies have specifically evaluated the effects of systemic LT supplementation on periodontal tissues or its influence on disease progression and therapeutic response. Therefore, the present study aimed to assess the impact of systemic LT supplementation on the progression of experimental periodontitis (EP) and on the outcomes of mechanical periodontal treatment in male rats, compared with a standard treatment group. The null hypothesis was that systemic LT supplementation does not reduce bone loss in the furcation region.

## 2. Materials and Methods

### 2.1. Animals

One hundred and twenty male rats (*Rattus norvegicus* albinus, Wistar), weighing 250–300 g and aged four months, were housed in a controlled environment (12/12 h light/dark cycle; temperature 22 ± 2 °C; 20 air changes per hour; relative humidity 55 ± 5%), with free access to food and water. The animals were monitored daily. The experimental protocol was approved by the Animal Ethics Committee of the School of Dentistry, Araçatuba, São Paulo State University (#0300/2021—24 June 2021), and was conducted in accordance with the ARRIVE guidelines [29].

### 2.2. Sample Calculation and Randomization

The NC3Rs Reporting Guidelines Working Group, were followed for sample size determination. For the experimental sample size calculation, a statistical power of 80% and an alpha level of 5% were adopted, based on a minimum detectable difference of 10% and an assumed standard deviation of 10% between groups. These data were analyzed using BioEstat 5.0 (Sociedade Civil de Mamirauá, Belém, PA, Brazil), which indicated a minimum of eight repetitions per period/group, resulting in a total of 120 animals. The sample size calculation was based on de Almeida et al. (2019) [12], considering the percentage of bone in the furcation region as the primary outcome variable. This study employed a randomized, single-blind, controlled design. Simple randomization (1:1 allocation) was performed by an individual not involved in the experimental procedures. Animals’ tails were labelled from 1 to 120, and the numerical sequence was imported into Minitab^®^ 17 (Minitab Inc., State College, PA, USA) to generate a table of random numbers.

### 2.3. Experimental Groups

The animals were allocated into two primary experimental groups: a non-treatment group (NT), in which only the progression of experimental periodontitis (EP) was assessed, and a scaling and root planning group (SRP), in which periodontal treatment was performed. Each primary group was subsequently subdivided into vehicle (VEH) or lutein (LT) subgroups.

- **SRP/VEH group (*n* = 30):** EP was induced using a ligature, and animals received daily gastric gavage (GG) of 0.7 mL/kg VEH until the end of the experiment. Seven days after EP induction (designated as day 0 for the treatment groups), the ligature was removed and SRP was performed.
- **SRP/LT group (*n* = 30):** EP was induced using a ligature, and animals received daily supplementation of 250 mg/kg LT via GG until the end of the experiment. Seven days after EP induction (day 0), the ligature was removed and SRP was performed.
- **NT/VEH group (*n* = 30):** EP was induced using a ligature, and animals received daily GG of 0.7 mL/kg VEH until the end of the experiment.
- **NT/LT group (*n* = 30):** EP was induced using a ligature, and animals received daily supplementation of 250 mg/kg LT via GG until the end of the experiment.

### 2.4. Preparation of the LT

Lutein (LT) at 10% (Chenguang BioTech, Xinjiang, China—LTC30223201027; internal batch 038706), extracted from marigold flowers and classified as a plant-derived source (molecular weight: 568.9 g/mol), was incorporated into an inert oil-based vehicle (Iberoquímica Farmacêutica, Jundiaí, SP, Brazil—LT210401186) to obtain a final concentration of 250 mg/mL (Aphoticario Pharmaceutical Lab SA, Araçatuba, SP, Brazil). The shelf life of the formulation was estimated at 7 days after preparation.

The LT solution was prepared weekly by the manufacturer (Aphoticario Pharmaceutical Lab SA, Araçatuba, SP, Brazil) in quantities sufficient for administration throughout each experimental week. After preparation, the solution was stored in amber glass containers under refrigeration at 4 °C to minimize degradation. Due to the photosensitive nature of LT, all handling and administration procedures were conducted under reduced light conditions. Prior to each administration, the solution was gently homogenized by daily agitation. According to the certificate of analysis and analytical reference provided by the manufacturer, the formulation presented a yellow-orange coloration, loss on drying of 4.65% (maximum of 6%; 5 g at 105 °C for 2 h), and residue on ignition of 0.34% (maximum of 1%; 3 g at 550 °C for 4 h). Microbiological analysis confirmed the absence of total aerobic and anaerobic bacteria, as well as fungi. The total lutein content was 10.03%, quantified by high-performance liquid chromatography (HPLC).

### 2.5. Sedation and General Anesthesia

For all experimental procedures (EP and SRP), the animals were anaesthetized with a combination of ketamine hydrochloride (80 mg/kg; Francotar^®^, Virbac, Roseira, SP, Brazil) and xylazine hydrochloride (10 mg/kg; Rompum^®^, Bayer, RS, Brazil), administered intramuscularly into the bilateral biceps femoris muscles of the hind limbs.

### 2.6. Induction of Experimental Periodontitis (EP)

On day 0, in all experimental groups, EP was induced by a calibrated and blinded operator (J.M.d.A.) using a 24-gauge cotton ligature (Coats Corrente, São Paulo, SP, Brazil) placed around the left mandibular first molar with the aid of modified forceps. The ligatures were positioned subgingival and secured with a simple surgical knot in the gingival sulcus near the alveolar bone crest [30,31].

### 2.7. Protocol for Supplementation with LT or VEH

**Figure 1:**
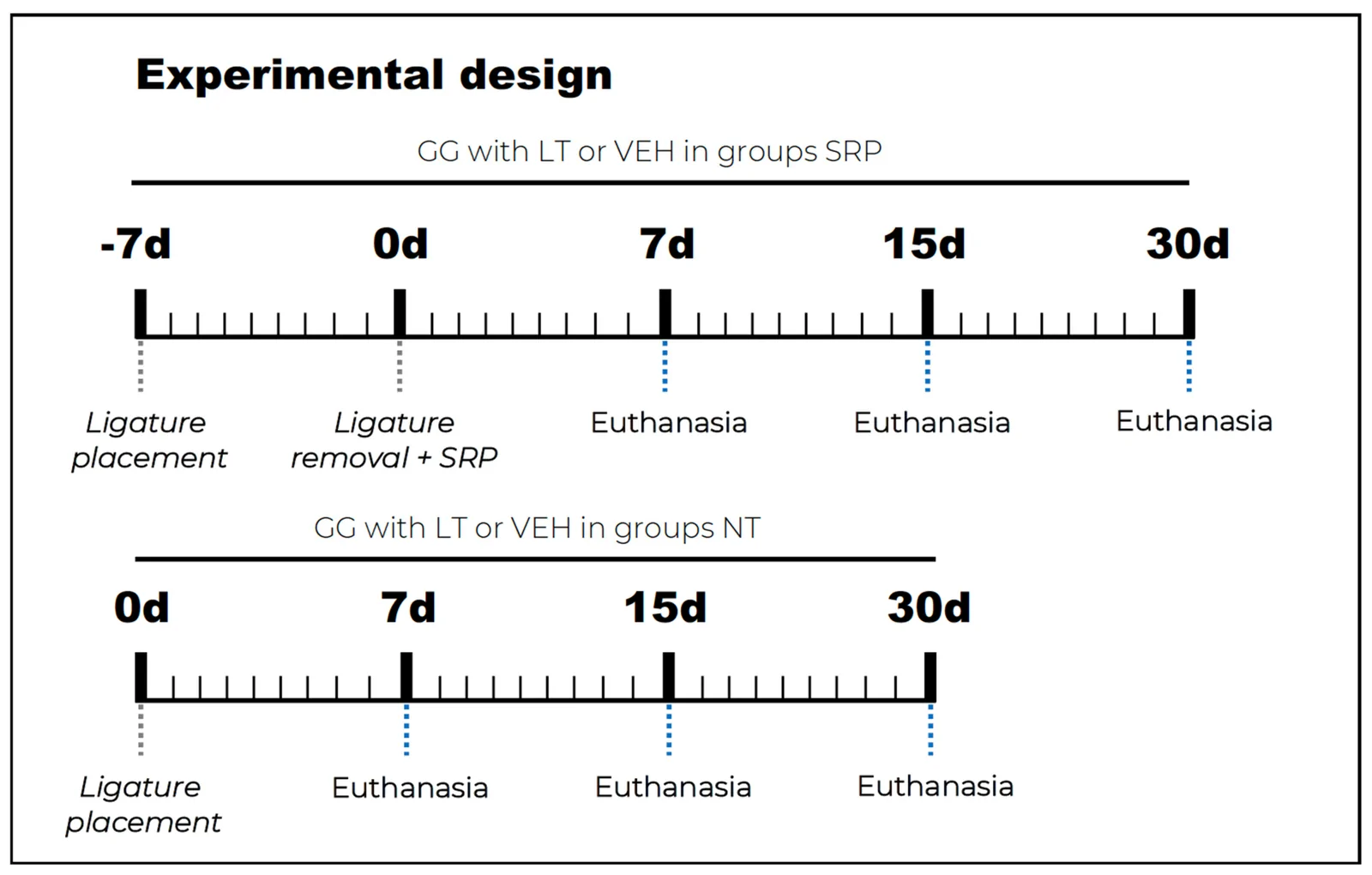
Experimental design.

Oral supplementation with VEH or LT was initiated on day 0 of EP induction for all experimental groups. Animals in the NT/LT and SRP/LT groups received LT supplementation at a dose of 250 mg/kg [32,33], whereas animals in the NT/VEH and SRP/VEH groups received 0.7 mL/kg of VEH to match the volume administered in the LT groups (Figure 1) [12].

### 2.8. Scaling and Root Planning Protocol

Scaling and root planning (SRP/VEH and SRP/LT) was performed by a blinded and calibrated researcher (J.M.d.A.), who was unaware of group allocation and experimental periods. Seven days after EP induction, the ligature was carefully removed using a clinical probe, and SRP was performed with 1–2 Minigracey hand curettes (Hu-Friedy, Chicago, IL, USA). Ten traction strokes were applied in a disto-mesial direction on the buccal and lingual surfaces, while the interproximal and furcation areas were instrumented using cervico-occlusal traction strokes [34].

### 2.9. Euthanasia and Processing

Animals were euthanized at 7, 15, and 30 days after EP induction (NT/VEH and NT/LT) and at 7, 15, and 30 days after periodontal treatment (SRP/VEH and SRP/LT), under strict technical and ethical oversight. Euthanasia was performed chemically by administering an overdose of anesthetic. Subsequently, the left hemimandibles were harvested, fixed in 4% buffered formaldehyde solution (Sigma-Aldrich, St. Louis, MO, USA) for 48 h at room temperature, and then immersed in 10% buffered ethylenediaminetetraacetic acid (EDTA) solution (Sigma-Aldrich, St. Louis, MO, USA) for two months, with biweekly solution changes. The samples were dehydrated in a graded ethanol series (70°–80°–90°–95°–100° I–100° II–100° III GL; Êxodo Científica, Sumaré, SP, Brazil), cleared in xylene (Êxodo Científica, Sumaré, SP, Brazil), infiltrated, and embedded in high-melting-point paraffin (Code 2006538; CAQ Química, Diadema, SP, Brazil). From each block, 4 μm-thick histological sections were obtained from the furcation center in a buccolingual progression, parallel to the sagittal plane. Six equidistant sections were stained with Harris hematoxylin and eosin (H&E) for histopathological assessment and histometric quantification of the percentage of bone in the furcation (PBF). An additional ten sections (two for each marker) were prepared for immunohistochemical (IHC) analysis to detect tartrate-resistant acid phosphatase (TRAP), tumor necrosis factor-alpha (TNF-α), interleukin-1β (IL-1β), interleukin-10 (IL-10), and osteocalcin (OCN).

Indirect immunoperoxidase staining for IHC [35]. After deparaffinization and hydration, antigen retrieval was carried out using heat-induced epitope retrieval (HIER) by immersing slides in citrate buffer solution (Diva decloaker, Biocare Medical, Concord, CA, USA) in a pressure chamber (Decloaking Chamber, Biocare Medical, Concord, CA, USA) at 95 °C for 20 min. Endogenous peroxidase and nonspecific binding sites were blocked with 3% hydrogen peroxide (Sigma Aldrich, St. Louis, MO, USA) for 1 h and 1% bovine serum albumin (Sigma Aldrich, St. Louis, MO, USA) for 12 h, respectively. Sections were divided into five groups, each incubated with one of the following primary antibodies: anti-TNFα (1:100; orb11495; Biorbyt, Cambridge, UK), anti-IL-1β (1:100; orb382131; Biorbyt, Cambridge, UK), anti-IL-10 (1:100; orb221323; Biorbyt, Cambridge, UK), anti-OCN (1:100; orb259644; Biorbyt, Cambridge, UK), and anti-TRAP (1:200; orb2250; Biorbyt, Cambridge, UK). Signal amplification was achieved using universal biotinylated secondary antibodies (1:200; goat IgG + rabbit IgG + mouse IgG) followed by streptavidin conjugated with peroxidase (Universal Dako Labelled HRP Streptavidin–Biotin Kit, Dako Laboratories, CA, USA). Visualization of the reaction product was performed using the 3,3′-diaminobenzidine (DAB) chromogen (DAB Chromogen Kit, Dako Laboratories, Carpinteria, CA, USA). Because immunostaining analysis was based on optical density, only slides stained for TRAP were counterstained with Harris hematoxylin to enable adequate visualization of tissue cytoarchitecture. All slides were subsequently dehydrated in ethanol (Êxodo Científica, Sumaré, SP, Brazil), cleared in xylene (Êxodo Científica, Sumaré, SP, Brazil), mounted with Permount (Fisher Scientific, San Diego, CA, USA), and coverslipped with glass coverslips (Olen, Kasvi, Pinhais, PR, Brazil).

### 2.10. Analysis of Results

**Figure 2:**
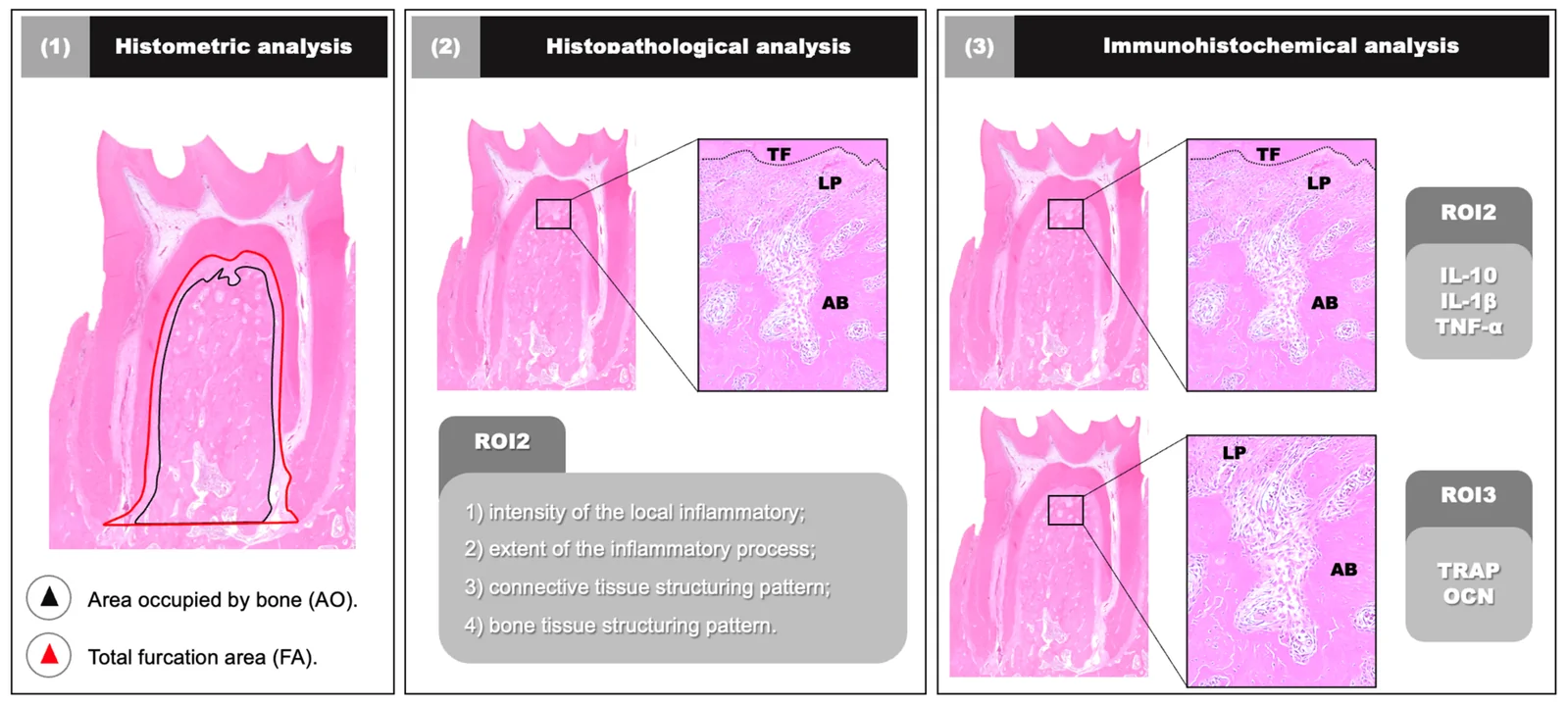
Analyses and regions of interest. Abbreviations: (TF) furcation roof; (AB) alveolar bone; (LP) periodontal ligament. Source: authors.

Histomorphometric, histopathological, and IHC analyses were performed by calibrated examiners who were blinded to the experimental groups and time points. A light microscope (Axio Scope, Carl Zeiss Microscopy GmbH, Oberkochen, Germany) equipped with a digital camera (AxioCam MRc5, Carl Zeiss Microscopy GmbH, Oberkochen, Germany) was used for image acquisition, and photomicrographs were captured using ZEN2 software (Version 3.2, Carl Zeiss Microscopy GmbH, Oberkochen, Germany). The following regions of interest (ROIs) were defined for analysis (Figure 2):

- **ROI1:** Total furcation area (FA), defined as the entire outline of the external surface of the cementum between the mesial and distal roots; the bone area (BA) corresponds to the entire outline of the external surface of the bone tissue between the roots. This ROI was used for histometric assessment of the PBF and for histological examination.
- **ROI2:** Furcation roof extending apically for 1000 μm towards the center of the interradicular septum, forming an area of 1000 × 1000 μm at 20× magnification. This ROI was used for IHC analysis of inflammatory markers (TNF-α, IL-1β, and IL-10).
- **ROI3:** External contour of the alveolar bone tissue in the furcation region, extending 1000 μm towards the center of the interradicular septum, forming an area of 1000 × 1000 μm at 20× magnification. This ROI was used for IHC analysis of bone markers (OCN and TRAP).

### 2.11. Histometric Analysis of the Percentage of Bone Tissue in the Furcation Region (PBF)

ROI1 was used to determine the PBF of the left mandibular first molar. Each specimen was measured three times, on three different days, by the same examiner (O.A.P.V.). ImageJ software (U.S. National Institutes of Health, Bethesda, MD, USA, https://imagej.net/ij/) was used with the “Polygon” tool (https://www.polygon.com) for delineation. The initial measurements of BA and FA were expressed in mm^2^. Subsequently, PBF was calculated by multiplying BA by 100 and dividing by FA (PBF = [BA × 100]/FA) [36].

### 2.12. Histopathological Analysis of the Furcation Area

A certified histologist (E.E.) evaluated the histopathological characteristics of the periodontal tissues in ROI2 using light optical microscopy (AxioLab^®^; Carl Zeiss Microscopy GmbH, Oberkochen, Germany). The parameters established by Zuza et al. (2018) [37], with modifications for the present study, were applied: (1) intensity of the local inflammatory response; (2) extent of the inflammatory process; (3) connective tissue structural pattern; and (4) bone tissue structural pattern. Each parameter was scored from 0 to 3 according to the histological features observed in each specimen.

### 2.13. Immunohistochemical Analysis

The optical density immunostaining technique was used to analyze images of immunolabelled sections [38]. ROI2 was used to assess the immunostaining density of TNF-α, IL-1β, and IL-10, whereas ROI3 was used for OCN and TRAP. The analysis of TNF-α, IL-1β, IL-10, and OCN was performed using the color threshold tool in ImageJ software (Rasband, W.S., ImageJ, U.S. National Institutes of Health, Bethesda, MD, USA), converting images to 32-bit greyscale and determining density using the “Threshold” function. Immunostaining density was expressed as mean percentage and standard deviation. For the quantification of TRAP-positive cells, only immunoreactive cells exhibiting dark brown staining, visible nuclei, cytoplasmic localization, and direct contact with bone tissue were considered. Results were expressed as mean and standard deviation. The IHC analysis for each specimen was performed three times, on three different days, by the same examiner (O.A.P.V.).

### 2.14. Primary and Secondary Outcome

The primary outcome was defined as the percentage of bone in the furcation (PBF), assessed in ROI1. Secondary outcomes included histopathological characteristics and the density of immunostaining within their respective regions of interest.

### 2.15. Statistical Analysis

Data were statistically analyzed using BioStat software (version 5.0, Belém, PA, Brazil). The normal distribution of the data was assessed using the Shapiro–Wilk test. Nonparametric data were analyzed with the Friedman test followed by Dunn’s multiple comparison post hoc test (*p* ≤ 0.05). For parametric data, two-way ANOVA was employed, followed by Tukey’s post hoc test (*p* ≤ 0.05). The graphical representations were prepared according to the normality results obtained from the Shapiro–Wilk test. Scatter plots with error bars were used for parametric data, whereas boxplots with dispersion were used for non-parametric data. Intra-examiner calibration was assessed using Cohen’s kappa coefficient (κ) and the intraclass correlation coefficient (ICC).

## 3. Results

No complications were observed throughout the experimental period, and all animals remained in good health. Two animals from the SRP/VEH 30-day group were excluded due to loss of the ligature; these animals were replaced to avoid compromising the sample size and the replacement was performed during the experimental period. Intra-examiner calibration was confirmed using Cohen’s kappa coefficient (κ = 0.89) and the intraclass correlation coefficient (ICC = 0.91).

### 3.1. Histological and Histomorphometric Analysis

**Figure 3:**
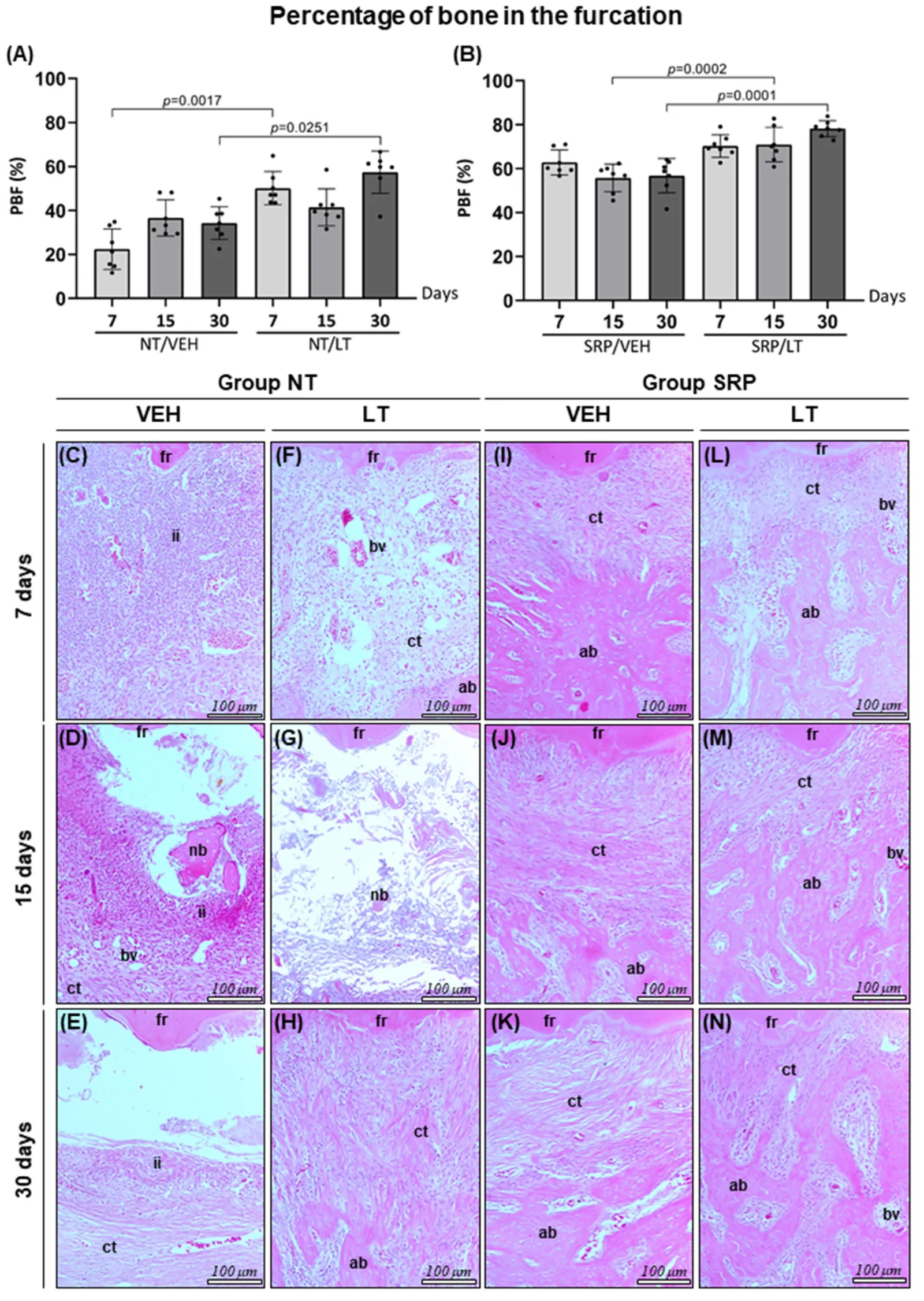
(**A**) Graph showing the mean, standard deviation, and distribution of the percentage of bone tissue in ROI1 of the NT group. (**B**) Graph showing the mean, standard deviation, and distribution of the percentage of bone tissue in ROI1 of the SRP group. (**C**–**H**) Photomicrographs showing bone tissue in the furcation region of the left mandibular first molar in the groups NT/VEH 7d (**C**), NT/VEH 15d (**D**), NT/VEH 30d (**E**), NT/LT 7d (**F**), NT/LT 15d (**G**), and NT/LT 30d (**H**). (**I**–**N**) Photomicrographs showing bone tissue in the same region in the groups SRP/VEH 7d (**I**), SRP/VEH 15d (**J**), SRP/VEH 30d (**K**), SRP/LT 7d (**L**), SRP/LT 15d (**M**), and SRP/LT 30d (**N**). Abbreviations: (fr) furcation roof; (ii) inflammatory infiltrate; (ct) connective tissue; (bv) blood vessel; (ab) alveolar bone; (nb) necrotic bone.

Specimens from the SRP/LT group, particularly at 30 days, exhibited reduced intensity and extent of the inflammatory process compared with the SRP/VEH group, characterised by the presence of few inflammatory cells. In addition, greater connective tissue organisation was observed in the furcation region, evidenced by a moderate number of fibroblasts and increased collagen fibre density, consistent with the formation of dense connective tissue. In both groups subjected to SRP (SRP/LT and SRP/VEH), irregular bone trabeculae were identified, associated with the presence of active osteoblasts and osteoclasts on the bone surface in most specimens analysed (Figure 3; Table S1—Supplementary Material).

In the NT/LT group, a progressive reduction in the inflammatory response was observed over the experimental periods, although partial compromise of the connective tissue persisted, characterised by a low number of collagen fibres and fibroblasts. A small number of active osteoclasts and irregularly contoured bone trabeculae were also noted. In contrast, specimens from the NT/VEH group displayed areas of necrotic bone tissue, increased numbers of active and inactive osteoclasts on the bone surface, and severe tissue disorganisation with extensive regions of necrosis (Figure 3; Table S1—Supplementary Material).

In the intergroup analysis, the NT/VEH group demonstrated lower PBF values indicating greater bone loss in the furcation region at 7 and 30 days (22.37 ± 9.18; 34.21 ± 7.42) compared ith the NT/LT group (50.15 ± 7.55; 57.38 ± 9.60) at the same experimental periods. Similarly, the SRP/VEH group showed lower PBF at 15 and 30 days (55.68 ± 6.28; 56.74 ± 7.78) compared with the SRP/LT group (70.22 ± 5.14; 78.17 ± 3.61). No statistically significant differences were observed in the intragroup comparisons at any experimental time point (Figure 3).

### 3.2. Immunohistochemical Analysis

#### 3.2.1. Immunolabeling Density for IL-1β and TNF-α

**Figure 4:**
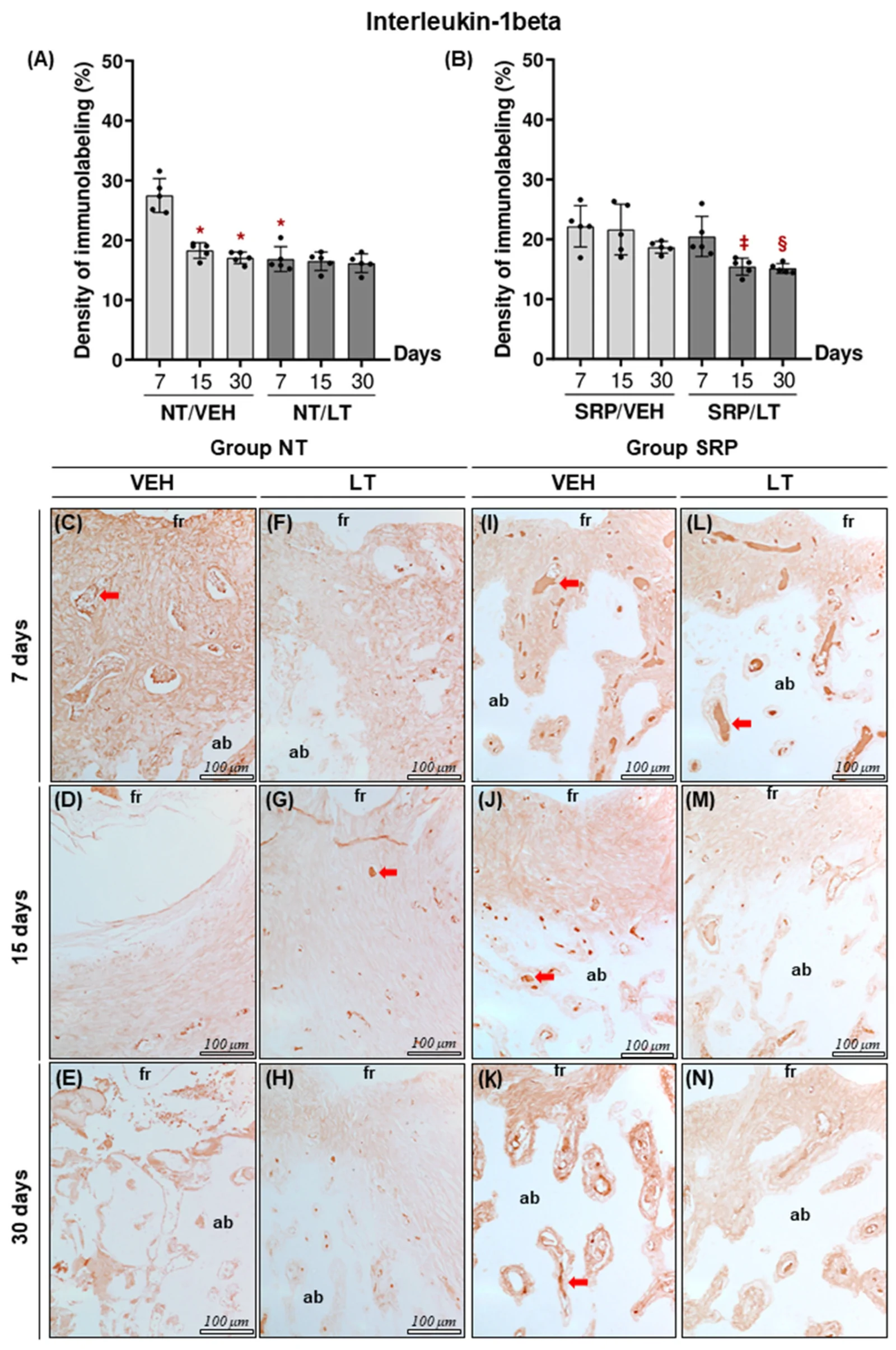
(**A**) Graph showing the mean, standard deviation, and distribution of the percentages of immunolabelling for IL-1β in ROI2 of the NT group. (**B**) Graph showing the mean, standard deviation, and distribution of the percentages of immunolabelling for IL-1β in ROI2 of the SRP group. (**C**–**H**) Photomicrographs showing the patterns of immunolabelling density for IL-1β in the furcation region of the lower first molar in the groups NT/VEH 7d (**C**), NT/VEH 15d (**D**), NT/VEH 30d (**E**), NT/LT 7d (**F**), NT/LT 15d (**G**), and NT/LT 30d (**H**). (**I**–**N**) Photomicrographs showing the patterns of immunolabelling density for IL-1β in the furcation region of the lower first molar in the groups SRP/VEH 7d (**I**), SRP/VEH 15d (**J**), SRP/VEH 30d (**K**), SRP/LT 7d (**L**), SRP/LT 15d (**M**), and SRP/LT 30d (**N**). Abbreviations: (fr) upper furcation region; (ab) alveolar bone. Symbols: (*) statistically significant difference compared with NT/VEH 7d; (‡) statistically significant difference compared with SRP/VEH 15d; (§) statistically significant difference compared with SRP/LT 7d. Red arrow: immunolabeling.

In the NT/VEH group, IL-1β immunolabeling density was higher at 7 days (27.49 ± 2.82) compared with 15 days (18.29 ± 1.31) and 30 days (17.05 ± 0.97) (*p* = 0.0001). In the SRP/LT group, IL-1β immunolabeling density was also higher at 7 days (20.46 ± 3.35) compared with 30 days (15.13 ± 0.81) (*p* = 0.0457). In the intergroup comparisons, the NT/VEH group showed higher IL-1β immunolabeling density than the NT/LT group at 7 days (27.49 ± 2.82 vs. 16.85 ± 2.09), while at 15 days the SRP/VEH group presented higher values than the SRP/LT group (21.62 ± 4.23 vs. 15.42 ± 1.41; *p* = 0.0150) (Figure 4).

**Figure 5:**
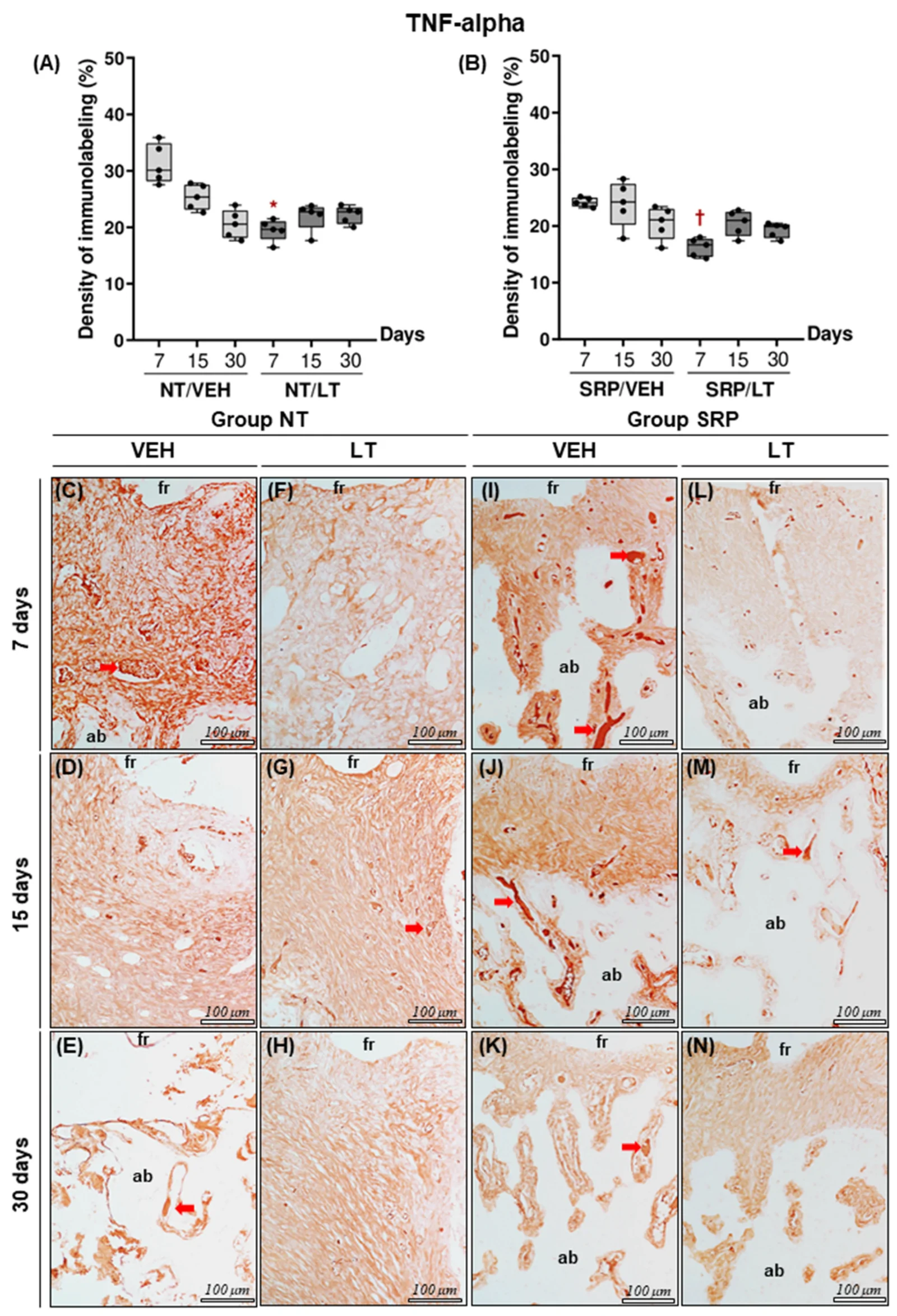
(**A**) Graph showing the mean, standard deviation, interquartile range, and distribution of the percentages of immunolabelling for TNFα in ROI2 of the NT group. (**B**) Graph showing the means, standard deviation, interquartile range, and distribution of the percentages of immunolabelling for TNFα in ROI2 of the SRP group. (**C**–**H**) Photomicrographs showing the patterns of immunolabelling density for TNFα in the furcation region of the lower first molar in the groups NT/VEH 7d (**C**), NT/VEH 15d (**D**), NT/VEH 30d (**E**), NT/LT 7d (**F**), NT/LT 15d (**G**), and NT/LT 30d (**H**). (**I**–**N**) Photomicrographs showing the patterns of immunolabelling density for TNFα in the furcation region of the lower first molar in the groups SRP/VEH 7d (**I**), SRP/VEH 15d (**J**), SRP/VEH 30d (**K**), SRP/LT 7d (**L**), SRP/LT 15d (**M**), and SRP/LT 30d (**N**). Abbreviations: (fr) upper furcation region; (ab) alveolar bone. Symbols: (*) statistically significant difference compared with NT/VEH 7d; (†) statistically significant difference compared with SRP/VEH 7d. Red arrow: immunolabeling.

For TNF-α, the NT/VEH (31.24 ± 3.52) and SRP/VEH (24.19 ± 0.81) groups exhibited higher immunolabelling density compared with the NT/LT (19.50 ± 1.92; *p* = 0.0030) and SRP/LT (16.24 ± 1.63; *p* = 0.0058) groups, respectively, at 7 days. No statistically significant intragroup differences were observed for TNF-α at any experimental time point (Figure 5).

#### 3.2.2. Immunolabeling Density for IL-10

**Figure 6:**
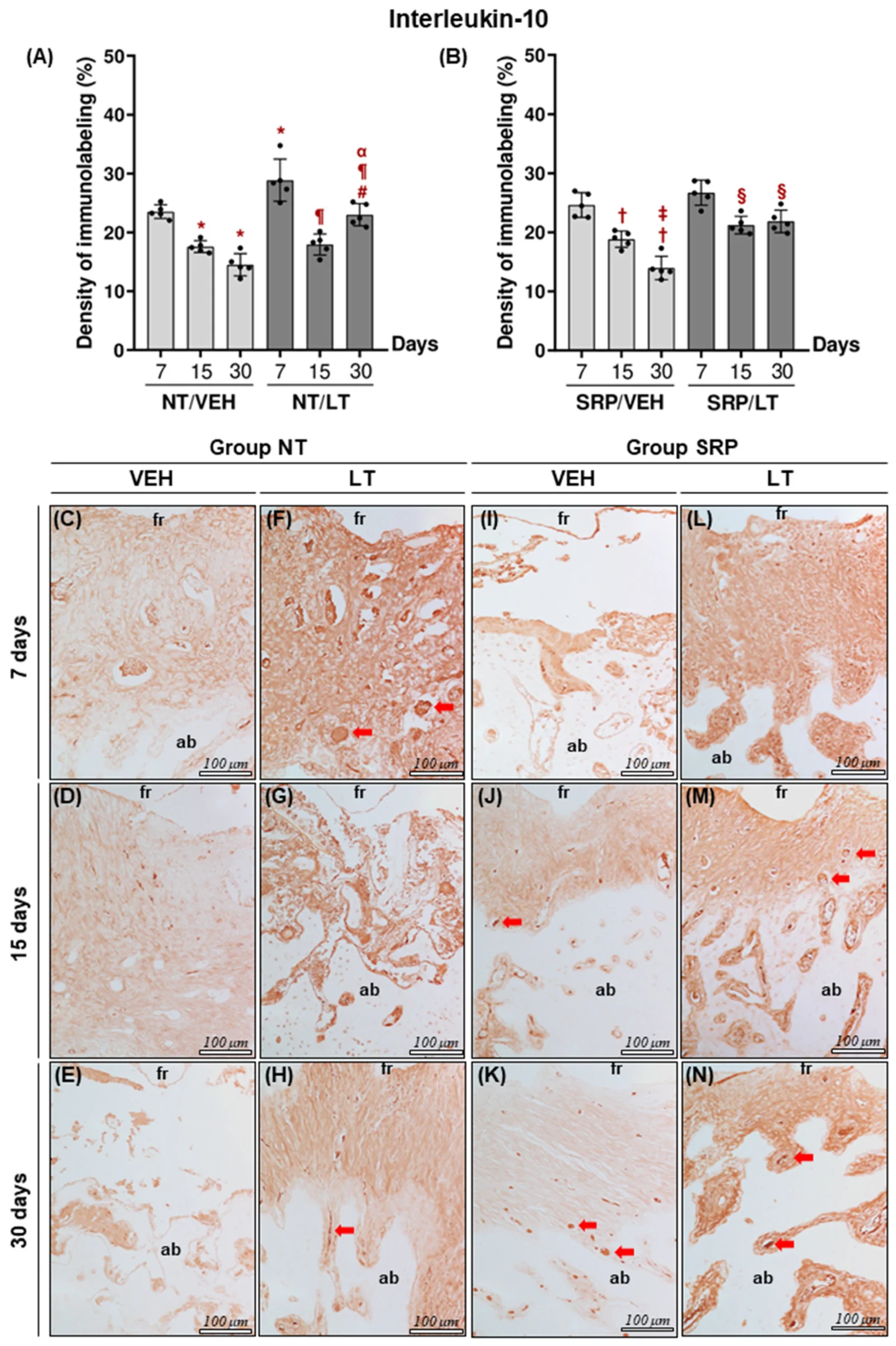
(**A**) Graph showing the mean, standard deviation, and distribution of the percentages of immunolabelling for IL-10 in ROI2 of the NT group. (**B**) Graph showing the means, standard deviation, and distribution of the percentages of immunolabelling for IL-10 in ROI2 of the SRP group. (**C**–**H**) Photomicrographs showing the patterns of immunolabelling density for IL-10 in the furcation region of the lower first molar in the groups NT/VEH 7d (**C**), NT/VEH 15d (**D**), NT/VEH 30d (**E**), NT/LT 7d (**F**), NT/LT 15d (**G**), and NT/LT 30d (H). (**I**–**N**) Photomicrographs showing the patterns of immunolabelling density for IL-10 in the furcation region of the lower first molar in the groups SRP/VEH 7d (**I**), SRP/VEH 15d (**J**), SRP/VEH 30d (**K**), SRP/LT 7d (**L**), SRP/LT 15d (**M**), and SRP/LT 30d (**N**). Abbreviations: (fr) upper furcation region; (ab) alveolar bone. Symbols: (*) statistically significant difference compared with NT/VEH 7d; (†) statistically significant difference compared with SRP/VEH 7d; (¶) statistically significant difference compared with NT/LT 7d; (α) statistically significant difference compared with NT/LT 15d; (‡) statistically significant difference compared with SRP/VEH 15d; (§) statistically significant difference compared with SRP/LT 7d; (#) statistically significant difference compared with NT/VEH 30d. Red arrow: immunolabeling.

In the intragroup analysis, the NT/VEH (23.52 ± 1.17) and NT/LT (28.87 ± 3.59) groups showed higher IL-10 immunolabelling density at 7 days compared with 15 and 30 days. In the NT/VEH group, values decreased at 15 days (17.56 ± 1.01; *p* = 0.0016) and 30 days (14.50 ± 1.90; *p* = 0.001), while in the NT/LT group, lower values were observed at 15 days (17.93 ± 1.79; *p* = 0.001) and 30 days (23.00 ± 1.88; *p* = 0.001). A similar pattern was observed in the SRP groups. The SRP/VEH (24.62 ± 2.07) and SRP/LT (26.70 ± 2.12) groups exhibited higher IL-10 immunolabelling density at 7 days compared with later time points. In the SRP/VEH group, values decreased at 15 days (18.84 ± 1.98; *p* = 0.0009) and 30 days (13.97 ± 1.98; *p* = 0.001), whereas in the SRP/LT group, reduced values were observed at 15 days (21.21 ± 1.50; *p* = 0.0016) and 30 days (21.85 ± 1.91; *p* = 0.0054). Additionally, an overall increase in IL-10 immunolabelling density was observed at 15 days (Figure 6).

#### 3.2.3. Immunolabeling Density for OCN

**Figure 7:**
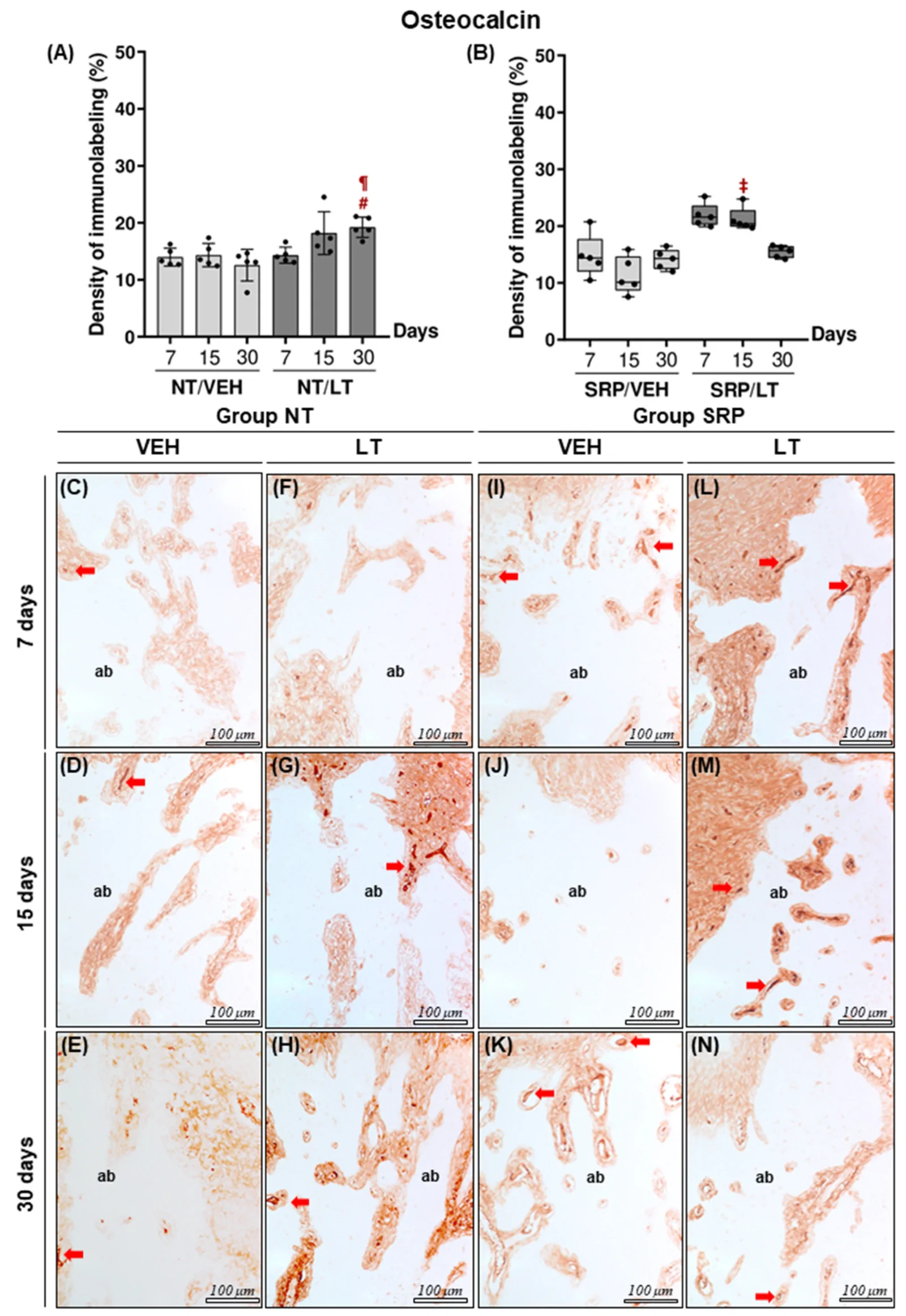
(**A**) Graph showing the mean, standard deviation, and distribution of the percentages of immunolabelling for OCN in ROI3 of the NT group. (**B**) Graph showing the means, standard deviation, interquartile ranges, and distribution of the percentages of immunolabelling for OCN in ROI3 of the SRP group. (**C**–**H**) Photomicrographs showing the patterns of immunolabelling density for OCN in the groups NT/VEH 7d (**C**), NT/VEH 15d (**D**), NT/VEH 30d (**E**), NT/LT 7d (**F**), NT/LT 15d (**G**), and NT/LT 30d (**H**). (**I**–**N**) Photomicrographs showing the patterns of immunolabelling density for OCN in the groups SRP/VEH 7d (**I**), SRP/VEH 15d (**J**), SRP/VEH 30d (**K**), SRP/LT 7d (**L**), SRP/LT 15d (**M**), and SRP/LT 30d (**N**). Abbreviations: (ab) alveolar bone. Symbols: (#) statistically significant difference compared with NT/VEH 30d; (¶) statistically significant difference compared with NT/LT 7d; (‡) statistically significant difference compared with SRP/VEH 15d. Red arrow: immunolabeling.

In the intragroup analysis, the NT/LT group exhibited lower OCN immunolabelling density at 7 days (14.40 ± 1.62; *p* = 0.0346) compared with 30 days (19.23 ± 1.79). In the intergroup analysis, the SRP/VEH group at 15 days (11.14 ± 3.29; *p* = 0.0352) and the NT/VEH group at 30 days (12.57 ± 2.78; *p* = 0.0025) showed lower OCN immunolabelling density compared with the SRP/LT (21.33 ± 2.32) and NT/LT (19.23 ± 1.79) groups at the corresponding time points, respectively. The OCN immunolabelling density values and statistical comparisons among the experimental groups are presented in Figure 7.

#### 3.2.4. Quantification of TRAP-Positive Cells/mm^2^

**Figure 8:**
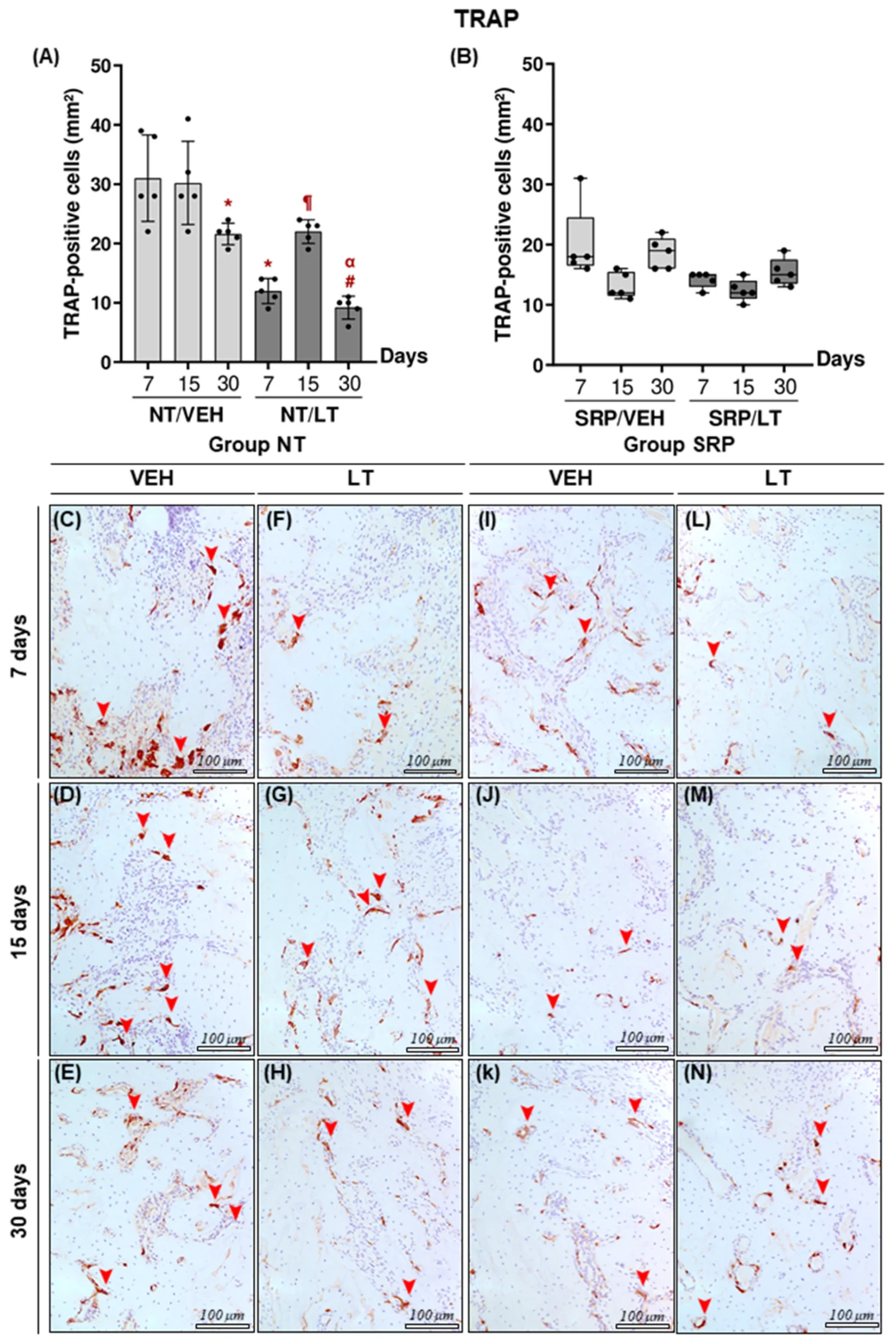
(**A**) Graph showing the mean, standard deviation, and distribution of the quantification of TRAP-positive cells (mm^2^) in ROI3 of the NT group. (**B**) Graph showing the means, standard deviation, interquartile ranges, and distribution of the quantification of TRAP-positive cells (mm^2^) in ROI3 of the SRP group. (**C**–**H**) Photomicrographs showing TRAP-positive cells in the furcation region in the groups NT/VEH 7d (**C**), NT/VEH 15d (**D**), NT/VEH 30d (**E**), NT/LT 7d (**F**), NT/LT 15d (**G**), and NT/LT 30d (**H**). (**I**–**N**) Photomicrographs showing TRAP-positive cells in the furcation region in the groups SRP/VEH 7d (**I**), SRP/VEH 15d (**J**), SRP/VEH 30d (**K**), SRP/LT 7d (**L**), SRP/LT 15d (**M**), and SRP/LT 30d (**N**). Symbols: (#) statistically significant difference compared with NT/VEH 30d; (¶) statistically significant difference compared with NT/LT 7d; (*) statistically significant difference compared with NT/VEH 7d; (α) statistically significant difference compared with NT/LT 15d. Red arrow: immunolabeling.

In the intragroup analysis, the NT/VEH group exhibited a higher number of TRAP-positive cells/mm^2^ at 7 days (31.0 ± 7.28; *p* = 0.0476) and 15 days (30.20 ± 7.01; *p* = 0.001) compared with 30 days (21.60 ± 1.82). In the NT/LT group, the number of TRAP-positive cells/mm^2^ was higher at 15 days (22.0 ± 2.00) compared with both 7 days (12.0 ± 2.00; *p* = 0.0306) and 30 days (20.0 ± 1.92; *p* = 0.0038). No statistically significant intra- or intergroup differences were observed for the SRP/VEH and SRP/LT groups at any experimental time point. The quantification of TRAP-positive cells and corresponding statistical comparisons among the experimental groups are presented in Figure 8.

## 4. Discussion

Studies in the literature have demonstrated that LT exhibits therapeutic potential capable of modulating the pathogenesis of a variety of immunoinflammatory diseases, including periodontitis [39]. Accordingly, the present study evaluated the influence of systemic LT supplementation on the progression of experimental periodontitis and its impact on mechanical treatment. Based on the findings obtained, the null hypothesis was rejected, as systemic LT supplementation reduced bone loss. In addition, the results showed that SRP, when combined with systemic LT supplementation, attenuated the intensity and extent of the inflammatory infiltrate, decreased bone tissue destruction, as evidenced by a greater percentage of bone in the furcation region through histomorphometric analysis and preserved connective tissue integrity in the animal model used.

The use of experimental periodontitis models is essential for understanding the characteristics and molecular mechanisms associated with the immunopathogenesis of periodontal disease. Among the available methods, ligature-induced periodontitis, either alone or in combination with lipopolysaccharide (LPS) application and/or inoculation of periodontopathogenic microorganisms, primarily *Porphyromonas gingivalis*, is widely employed in periodontal research. It is well established that ligature-induced periodontitis promotes disruption of the epithelial and connective tissue integrity as a consequence of intensified local proinflammatory responses [40,41]. Previous studies have shown that experimental periodontitis presents two distinct phases of alveolar bone loss progression and immunoinflammatory response [42,43]. Up to 14 days after ligature placement, tissue destruction progresses alongside intensification of the local inflammatory infiltrate, particularly marked by increased gene expression of proinflammatory cytokines (TNF-α, IL-1β, and IL-6). After this period, a transition towards a chronic stage occurs, characterized by persistence of the inflammatory process and subsequent spontaneous tissue repair up to approximately 30 days. These findings are consistent with those of the present study, in which stabilization of the destructive process was observed in control groups over a similar period. Moreover, the ligature-induced periodontitis model in rodents is well consolidated in the literature, as rodents exhibit morphophysiological features and immunoinflammatory responses comparable to those observed in human periodontitis [40].

As highlighted previously, the NF-κB signaling pathway is widely recognized for its role in the expression of chemokines and pro-inflammatory cytokines associated with periodontal tissue damage [44]. Previous studies have demonstrated that lutein (LT) significantly reduced the expression of TNF-α and IL-1β, concomitantly with decreased NF-κB protein levels, in a monosodium iodoacetate-induced osteoarthritis model in primary chondrocytes [45]. These findings are further supported by evidence showing that LT reduced NF-κB, TNF-α, and IL-1β levels in differentiated adipocytes and RAW264 cells induced by adipogenesis, due to the suppression of IKKα/β phosphorylation, a regulatory kinase essential for NF-κB transcriptional activation [46,47]. Additionally, LT attenuated neuroinflammation in LPS-stimulated BV2 microglial cells, partly through inhibition of NF-κB activation [48]. The aforementioned studies highlight the role of LT in cellular pathways and in the modulation of pro-inflammatory cytokines directly related to the immunopathogenesis of periodontitis, although investigated under different experimental conditions. Notably, and consistent with our findings, systemic LT administration significantly reduced immunolabelling for TNF-α and IL-1β compared with respective controls, particularly at later time points (15 and 30 days). Further in vivo studies reinforce the anti-inflammatory properties of LT, reporting reduced TNF-α levels in ovariectomized rats systemically supplemented with LT [49], following a protocol similar to ours, while other authors observed decreased IL-1β and TNF-α expression in a murine model of auricular inflammation induced by *Propionibacterium acnes* [50]. Taken together, these findings suggest that LT may modulate the host immunoinflammatory response in an infected microenvironment by inhibiting or suppressing NF-κB activation and, consequently, reducing the expression of pro-inflammatory mediators such as TNF-α and IL-1β in immunoinflammatory pathological conditions, including those arising from dysbiosis, such as periodontitis. Such modulation may favor periodontal tissue homeostasis and attenuate tissue breakdown, positioning LT as a promising adjunctive therapeutic strategy to enhance mechanical periodontal treatment, considering that SRP reduces subgingival periodontopathogenic biofilm, decreases the local burden of pro-inflammatory cytokines (e.g., TNF-α, IL-1β, and IL-6) [51], and promotes a microenvironment conducive to periodontal repair [52]. This effect was corroborated by our results, in which the SRP/LT groups showed lower immunolabelling density for TNF-α and IL-1β at 15 and 30 days compared with controls, reinforcing the potential of LT as an adjunct in the treatment of experimental periodontitis. However, it is important to emphasize that these interpretations are based on previously published evidence and represent mechanistic inferences intended to contextualize the present findings. The signaling pathways involved, such as NF-κB, were not directly evaluated in this study.

Phytochemicals have been extensively investigated as adjuncts to periodontal therapy, aiming to improve treatment predictability due to their pharmacological properties (e.g., anti-inflammatory, antioxidant, and tissue-repair-promoting activities), particularly in cases involving morphological deficiencies at the treated site or specific host-related factors [53]. Among these compounds, LT has shown potential to act on key processes in bone metabolism, favoring mineralized tissue repair, which is a relevant aspect in the treatment of periodontitis. It has been demonstrated that LT suppresses IL-1-induced bone resorption in ex vivo mouse cultures by inhibiting TRAP-positive osteoclasts [25]. LT also reduced IL-1-induced RANKL and sclerostin mRNA expression in primary osteoblasts, while increasing BMP2 mRNA expression and stimulating mineralized nodule formation. In addition, LT inhibited the differentiation of RAW264.7 cells into osteoclasts and induced apoptosis of these cells in the absence of RANKL. Complementarily, other studies have shown in vitro that LT promotes mineralized nodule formation in osteoblast cultures and reduces 1α,25-dihydroxyvitamin D_3_-induced osteoclast formation, while systemic LT supplementation in mice increased femoral bone mineral density [26]. Consistent with these findings, our results demonstrated positive effects of systemic LT administration, particularly when combined with SRP, including greater bone presence in the furcation region, increased OCN immunolabelling, and a reduced number of TRAP-positive multinucleated cells compared with controls. These findings suggest a shift in bone metabolism toward a reparative profile, characterized by enhanced osteoblastic activity and reduced osteoclastic resorption. Histopathological analysis corroborated these observations by demonstrating viable bone tissue and active osteoblasts on bone surfaces in LT-treated groups, especially in animals subjected to SRP. However, despite these promising results, the precise mechanisms underlying these effects in the context of periodontitis remain to be fully elucidated.

IL-10 is a key anti-inflammatory cytokine produced by various cell types, including lymphocytes, macrophages, and epithelial cells [54]. It plays a significant role in the immunopathogenesis of periodontitis by supporting bone homeostasis, promoting osteoblast differentiation, and inhibiting osteoclastogenesis [55]. Studies have demonstrated that LT, at high concentrations, downregulated Nrf2 and NF-κB mRNA expression while increasing the synthesis and release of IL-10 and TGF-β1 [56]. Similarly, other authors have reported increased IL-10 mRNA levels in birds supplemented with LT and exposed to *Salmonella typhimurium* LPS [57]. In addition, LT exposure in BV2 microglial cells significantly increased IL-10 secretion [58]. In the present study, LT-treated groups exhibited higher IL-10 immunolabelling, particularly at 7 and 30 days, compared with their respective control groups under the experimental conditions employed. It is important to note that the experimental models described in the literature as a theoretical basis differ substantially from the design adopted in the present study, which limits the direct comparability of the findings and requires cautious interpretation.

This study presents promising results regarding the potential applicability of LT as a systemic modulatory approach for the treatment of periodontitis. Nonetheless, certain methodological limitations warrant consideration to refine future investigations. The pharmacokinetic properties of LT require careful attention, particularly regarding formulation strategies and administration methods in experimental models. Studies indicate that factors such as digestive efficiency and host nutritional status may compromise LT gastrointestinal absorption, affecting its bioavailability and bioaccessibility [59]. Despite its less favorable pharmacokinetic profile, systemic LT administration successfully controlled EP progression and significantly enhanced periodontal repair when combined with mechanical treatment. Given its lipophilic nature and low chemical stability, which reduce aqueous solubility and plasma concentrations, LT was diluted in an inert oil-based vehicle [60]. Additionally, only male rats were used in this study, without accounting for sex-related biological differences, which are known to influence periodontal disease pathogenesis. For example, Duan et al. (2016) [61] reported greater susceptibility and more rapid progression of periodontitis in female mice. For future research, we recommend the use of nanostructured systems, such as nanoemulsions or polymer–lipid hybrid nanoparticles, to enhance LT bioavailability and distribution in both local and systemic delivery approaches, as well as studies evaluating the potential of LT as a preventive agent in the management of periodontitis.

## 5. Conclusions

Systemic lutein supplementation reduced inflammation and alveolar bone resorption, enhanced the effects of mechanical periodontal therapy, and promoted improved regeneration of the periodontal supporting tissues.

## Data Availability

The datasets used and/or analyzed during the current study are available from the corresponding author upon reasonable request.

## Supplementary Materials

The following supporting information can be downloaded at: https://www.mdpi.com/article/10.3390/dj14080466/s1, Table S1: Parameters, scores, and distribution of specimens according to the histopathological analysis of periodontal tissues in the furcation region of the mandibular first molar across the experimental groups.

## Abbreviations

AB	Alveolar bone
BA	Bone area
BMP-2	Bone morphogenetic protein 2
BV	Blood vessel
BV2	BV2 microglial cell line
CAT	Carotenoids
CT	Connective tissue
EDTA	Ethylenediaminetetraacetic acid
EP	Periodontitis
FA	Total furcation area
FR	Furcation roof
GG	Gastric gavage
HIER	Heat-induced epitope retrieval
II	Inflammatory infiltrate
IL-1β	Interleukin 1 beta
IL-6	Interleukin 6
IL-8	Interleukin 8
IL-10	Interleukin 10
IL-12	Interleukin 12
LPS	Lipopolysaccharide
LT	Lutein
mRNA	Messenger ribonucleic acid
NB	Necrotic bone
NF-κB	Nuclear factor kappa B
Nrf2	Nuclear factor erythroid 2–related factor 2
OCN	Osteocalcin
PBF	Percentage of bone in the furcation
RANKL	Receptor activator of nuclear factor kappa B ligand
SRP	Scaling and root planing
TGF-β1	Transforming growth factor beta 1
TNF-α	Tumour necrosis factor alpha
TRAP	Tartrate-resistant acid phosphatase
VEH	Vehicle
