# Supplementary material for: Evaluation of Systemic Lutein Supplementation on Periodontal Repair in Experimental Periodontitis in Wistar Rats
Source: Dent J (Basel). 2026 Aug 2;14(8):466. doi: 10.3390/dj14080466 (PMC13511582; doi:10.3390/dj14080466)
Supplement: Supplementary file 1 [file dentistry-14-00466-s001.zip › dentistry-4169056-supplementary.pdf]

Table S1. Parameters, scores, and distribution of specimens according to the histopathological analysis of periodontal tissues in the furcation region of the mandibular first molar across the experimental groups.

HISTOPATHOLOGICAL ASSESSMENT												
**PARAMETERS AND CORRESPONDING SCORES**	**PERCENTAGE OF SPECIMENS**											
	**EXPERIMENTAL GROUPS**											
	**NT/VEH**			**NT/LT**			**SRP/VEH**			**SRP/LT**		
	**7d**	**15d**	**30d**	**7d**	**15d**	**30d**	**7d**	**15d**	**30d**	**7d**	**15d**	**30d**
**INTENSITY OF THE LOCAL INFLAMMATORY RESPONSE**												
**1)** Absence of inflammation	-	-	-	-	-	-	-	-	60%	-	-	80%
**2)** Mild inflammation (up to one-third of the cells are inflammatory)	-	-	-	-	-	-	20%	60%	40%	60%	100%	20%
**3)** Moderate inflammation (from one-third to two-thirds of the cells are inflammatory)	-	20%	40%	20%	40%	60%	80%	40%	-	40%	-	-
**4)** Severe inflammation (more than two-thirds of the cells are inflammatory)	100%	80%	60%	80%	60%	40%	-	-	-	-	-	-
**MEDIAN**	**4**	**4**	**4**	**4**	**4**	**3**	**3**	**2**	**1**	**2**	**2**	**1**
**EXTENT OF THE INFLAMMATORY PROCESS**												
**1)** Absence of inflammation	-	-	-	-	-	-	-	-	60%	-	-	80%
**2)** Extending into part of the connective tissue in the furcation region	-	-	-	-	-	-	40%	100%	40%	60%	100%	20%
**3)** Extending throughout the connective tissue in the furcation region	-	20%	40%	60%	60%	80%	60%	-	-	40%	-	-
**4)** Extending throughout both the connective tissue and the bone tissue in the furcation region	100%	80%	60%	40%	40%	20%	-	-	-	-	-	-
**MEDIAN**	**4**	**4**	**4**	**3**	**3**	**3**	**3**	**2**	**1**	**2**	**2**	**1**
**STRUCTURAL PATTERN OF THE CONNECTIVE TISSUE IN THE FURCATION REGION**												
**1)** Moderate number of fibroblasts and a large amount of collagen fibres (dense connective tissue)	-	-	-	-	-	-	-	-	60%	-	-	80%
**2)** Moderate numbers of both fibroblasts and collagen fibres	-	-	-	-	-	-	-	-	40%	-	-	20%
**3)** Small numbers of both fibroblasts and	40%	40%	40%	60%	60%	60%	80%	100%	-	80%	100%	-

	collagen fibres												
**4)**	Severe tissue disorganisation with areas of necrosis	60%	60%	60%	40%	40%	40%	20%	-	-	20%	-	-
	**MEDIAN**	**4**	**4**	**4**	**3**	**3**	**3**	**3**	**2**	**1**	**3**	**2**	**1**
**STRUCTURAL PATTERN OF THE BONE TISSUE IN THE FURCATION REGION**													
**1)**	Bone trabeculae with regular contours and numerous active osteoblasts, including areas of new bone formation	-	-	-	-	-	-	-	-	20%	-	-	20%
**2)**	Bone trabeculae with irregular contours and covered by both active osteoblasts and osteoclasts	-	-	-	-	-	-	100%	100%	80%	100%	100%	80%
**3)**	Bone trabeculae with irregular contours and numerous active osteoclasts	40%	40%	40%	60%	80%	80%	-	-	-	-	-	-
**4)**	Areas of necrotic bone and bone trabeculae with irregular contours containing numerous active and/or inactive osteoclasts	60%	60%	60%	40%	20%	20%	-	-	-	-	-	-
	**MEDIAN**	**4**	**4**	**4**	**3**	**3**	**3**	**2**	**2**	**2**	**2**	**2**	**2**
